# Impedance Changes and Fibrous Tissue Growth after Cochlear Implantation Are Correlated and Can Be Reduced Using a Dexamethasone Eluting Electrode

**DOI:** 10.1371/journal.pone.0147552

**Published:** 2016-02-03

**Authors:** Maciej Wilk, Roland Hessler, Kenneth Mugridge, Claude Jolly, Michael Fehr, Thomas Lenarz, Verena Scheper

**Affiliations:** 1 Department of Otolaryngology, Hannover Medical School, Hannover, Germany; 2 Clinic for Exotic Pets, Reptiles, Pet and Feral Birds, University of Veterinary Medicine, Foundation, Hannover, Germany; 3 MED-EL GmbH, Innsbruck, Austria; 4 Cluster of Excellence “Hearing4all”, Hannover Medical School, Hannover, Germany; Institute for Frontier Medical Sciences, Kyoto University, JAPAN

## Abstract

**Background:**

The efficiency of cochlear implants (CIs) is affected by postoperative connective tissue growth around the electrode array. This tissue formation is thought to be the cause behind post-operative increases in impedance. Dexamethasone (DEX) eluting CIs may reduce fibrous tissue growth around the electrode array subsequently moderating elevations in impedance of the electrode contacts.

**Methods:**

For this study, DEX was incorporated into the silicone of the CI electrode arrays at 1% and 10% (w/w) concentration. Electrodes prepared by the same process but without dexamethasone served as controls. All electrodes were implanted into guinea pig cochleae though the round window membrane approach. Potential additive or synergistic effects of electrical stimulation (60 minutes) were investigated by measuring impedances before and after stimulation (days 0, 7, 28, 56 and 91). Acoustically evoked auditory brainstem responses were recorded before and after CI insertion as well as on experimental days 7, 28, 56, and 91. Additionally, histology performed on epoxy embedded samples enabled measurement of the area of scala tympani occupied with fibrous tissue.

**Results:**

In all experimental groups, the highest levels of fibrous tissue were detected in the basal region of the cochlea in vicinity to the round window niche. Both DEX concentrations, 10% and 1% (w/w), significantly reduced fibrosis around the electrode array of the CI. Following 3 months of implantation impedance levels in both DEX-eluting groups were significantly lower compared to the control group, the 10% group producing a greater effect. The same effects were observed before and after electrical stimulation.

**Conclusion:**

To our knowledge, this is the first study to demonstrate a correlation between the extent of new tissue growth around the electrode and impedance changes after cochlear implantation. We conclude that DEX-eluting CIs are a means to reduce this tissue reaction and improve the functional benefits of the implant by attenuating electrode impedance.

## Introduction

The cochlear implant-electrode array consists of platinum-iridium and silicone. Both materials have a long tradition as implant materials and have proven excellent biocompatibility. However, both materials are recognized as a foreign body. Their implantation induces fibrotic capsule formation around the implant [1, 2]. In the case of cochlear implants this tissue casting is widely described [3–5] and is considered strongly to be the reason for post-operative increases of electrical impedance [6] and may lead to a reduction of channel separation. Such increases in electrode impedance during the first 2 to 4 weeks after implantation has been reported for recipients of various cochlear implant models [7–9] and a link between impedance changes and new tissue formation is suggested [6, 10] but as yet, not proven.

Higher impedances cause higher voltages generated across the electrode-tissue interface. This may also lead to a saturation of the current source and may therefore decrease the dynamic range of the stimulation. High electrode voltages lead to increased energy consumption resulting in shorter durability of the implant’s batteries. These issues have to be addressed especially in view of the economic outcome of today’s CI generation and for future technologies such as fully implantable CIs. Initial investigations in patient studies provide an indication that decreased impedance after cochlear implant surgery can be achieved by glucocorticoid application [11]. However, data obtained from the latter study were heterogeneously distributed probably as a result of inadequate control of the substance amount applied as well as the variable levels and distribution of the drug in the inner ear. Controlled drug delivery to the inner ear requires specially designed catheters if applied through injection [12].

To date, no drug delivery system exists which allows a localized specific treatment with a pharmacological entity to the inner ear or of the electrode-nerve-interface in order to reduce both the tissue response and increased impedance. Specifically, no commercial available device exists to ensure controlled local drug delivery for inner ear treatment which is combinable with the cochlear implant device.

Due to the nature of the inflammatory processes leading to implant encapsulation [13] glucocorticoids are considered as suitable agents to be administered locally to combat the over-shooting immune reaction. Corticoid receptor agonists such as DEX possess potent anti-inflammatory and anti-angiogenic properties and their influence on the immune system, connective tissue and fibroblasts, amongst others, has been demonstrated in a myriad of studies [14–16]. The effect of locally applied DEX on fibrotic capsule formation and impedance [17] has been reported diversely with some studies observing fibrous tissue reduction [18, 19] while others failed to see effect [20, 21]. The diversity of results may be explained by the high variability within experimental groups, insufficient levels of fibrotic tissue in the control groups or feasibly, to the inconsistent exposure times and concentrations of DEX presented to the cochlea.

The amount and duration of DEX to be applied is dependent on the drug delivery system used. In this study DEX-eluting electrode devices and non-drug loaded control devices were used to demonstrate; A) correlation between fibrous tissue growth and impedance increase; B) localized DEX treatment is sufficient to reduce fibrosis and electrode impedance; and C) the drug delivery system allows the application of a clinically relevant drug entity in amounts sufficient to produce effective therapeutic concentrations over an appropriate duration of time.

## Material and Methods

### Animals, experimental groups and anaesthesia

Female albino guinea pigs (Dunkin Hartley, Harlan) weighing between 350–390 g were used for this study. Animals were housed in groups of up to 5 individuals per cage. Animals are maintained in accordance with current regulations (German Animal Welfare Law, ETS123, Directive 2010/63/EU). All experiments are conducted in line with these regulations, are approved by the Local Institutional Animal Care and Research Advisory Committee of Hannover Medical School and permitted by the local authority (Lower Saxony State Office for Consumer Protection, Food Safety, and Animal Welfare Service, registration no. 10/0273).

The study used three randomly assigned groups based on two different concentrations of DEX (1% and 10%) in the CI’s silicone and a control group which were implanted with similarly processed electrodes without the active principal (hitherto termed 0% DEX). Animals (n = 27) were unilaterally implanted (left side) and received once weekly a 60 minute electrical stimulation. A subgroup (n = 6) in the 0% DEX group were additionally implanted on the contralateral side (right ear) with 0% DEX electrodes. These ears remained unstimulated apart from the stimulation pulse utilized during impedance measurements.

Anaesthesia was used prior to all surgical interventions as well as ABR and impedance measurements. A mixture of xylazine (10 mg/kg, Rompun^®^, Bayer, Germany) and ketamine (40 mg/kg, ketamin^®^, WDT, Germany) was administered by intramuscular (i.m) injection. As premedication, two drops of diazepam (Diazepam-ratiopharm^®^, Ratiopharm GmbH, Germany) were applied orally 30 minutes prior to surgery. All animals were given 0.05 mg/kg atropine sulfate subcutaneously (s.c) (Atropinsulfat^®^, B. Braun Melsungen AG, Germany) and 5 mg/kg carprofen s.c (Rimadyl^®^, Pfizer GmbH, Germany). Enrofloxacin (Baytril^®^, Bayer Animal Health GmbH, Germany) was given p.o on the day of surgery and for five consecutive days. Local anaesthesia with prilocaine (Xylonest^®^, AstraZeneca GmbH, Germany) was additionally applied before surgery in the mastoid area and on the craneum. If deemed necessary, animals received supplemental dosages to maintain anaesthesia. Following final measurements, all animals received a secondary administration of both local and systemic anaesthetics (15 minutes prior to euthanasia). A “pea-sized” portion of Bird Bene-Bac^®^ (Albrecht GmbH, Germany) was given to all animals one day prior to surgery, the day of surgery itself and the day after. Post-surgical surveillance of all animals was made daily for seven days by a veterinarian. Subsequently, animals were routinely checked daily by one of the housing staff and twice weekly by a veterinarian. Based on an in-house stress strategy which has been approved by the competent authority (LAVES), animals’ well-being was judged (Table 1). Animals with score 4 or 5 are directly euthanized if the therapy does not show a beneficial effect within four hours. Euthanasia is performed in anesthetized animals using T61 which is applied intracardially.

**Table 1:** Animal Health Score.

Score	Definition	Example
1	Normal well-being	No marks of pain; wound completely unremarkable
2	Marginally reduced behavior	Reduced feed intake, reduced activity, marginal reddening and swelling of the wound
3	Moderately reduced behavior	Minimal activity, expression of pain when wound is palpated, purulent wound
4	Severely changed behavior	Neurological deficits, refusal to eat, no faeces or urine
5	Critically changed behavior	Apathy

### Electrode design

**Fig 1:**
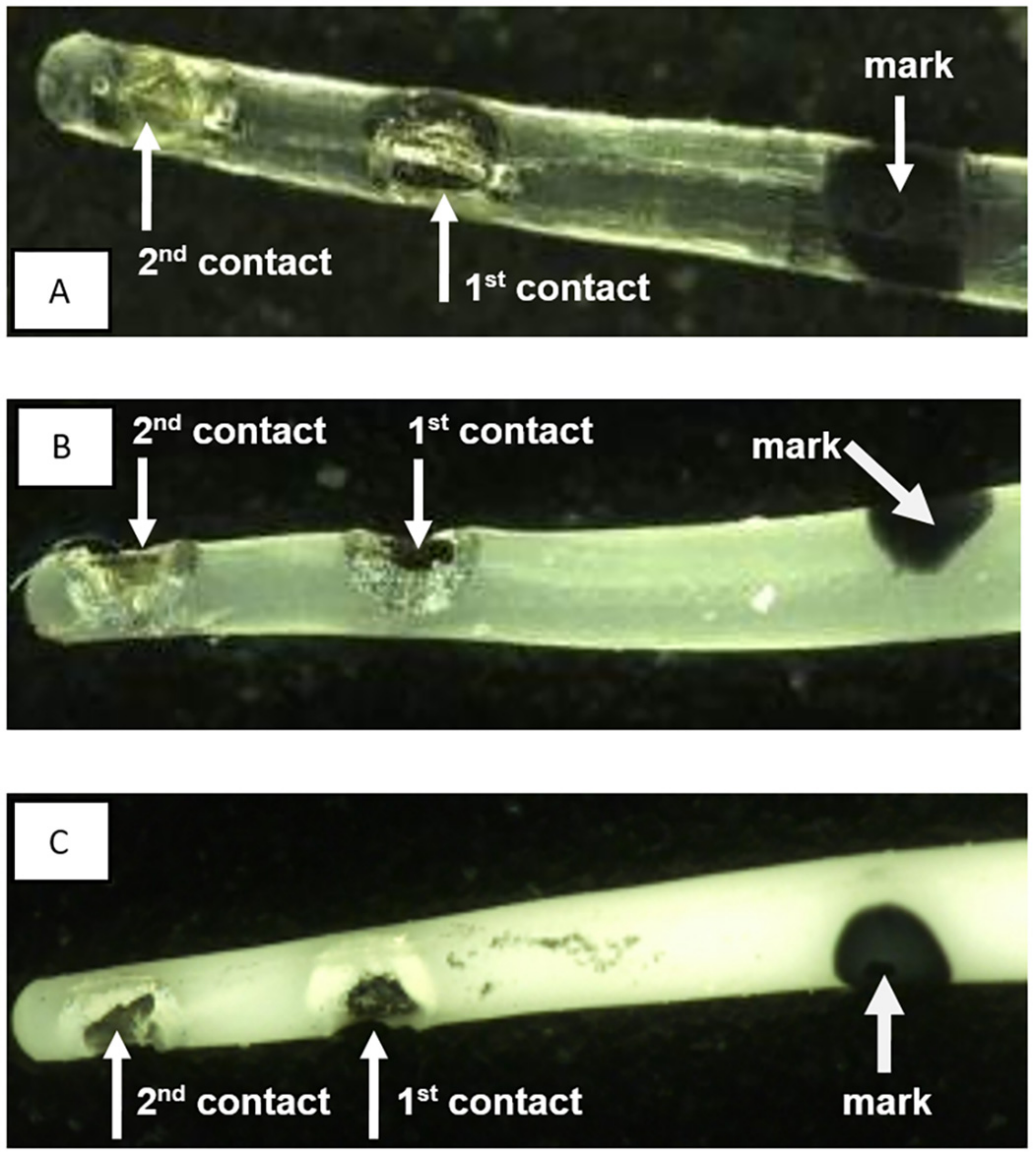
DEX eluting electrodes. The electrodes have two active contacts (1^st^ contact, more basal; 2^nd^ contact, more apical) and a black marker dot at 3 mm length from apex to indicate insertion depth; A: pure silicone electrode array without DEX (0%); B: electrode array containing 1% DEX (16 ng/day delivery rate); C: electrode array containing 10% DEX (49 ng/day delivery rate).

**Fig 2:**
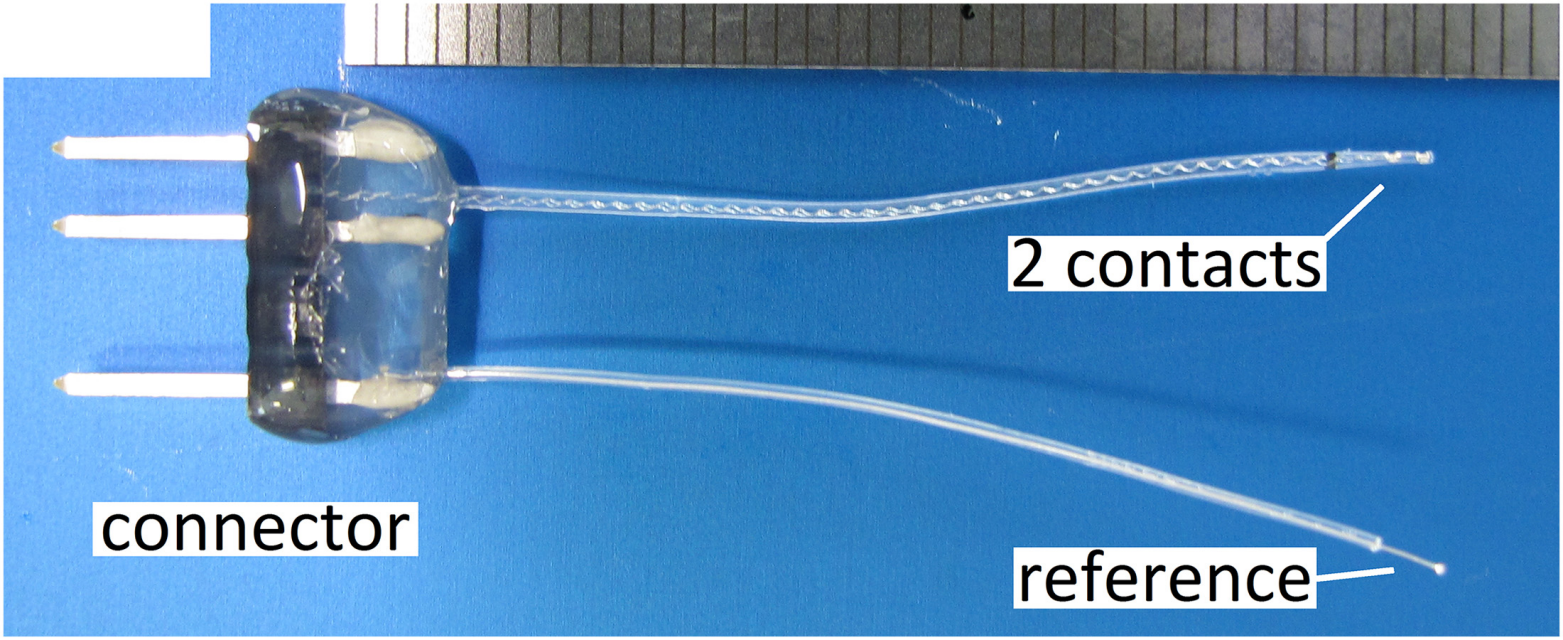
Guinea pig electrode. The electrodes used consist of two electrical contacts and a reference electrode. The connector was attached to the animal’s skull.

**Fig 3:**
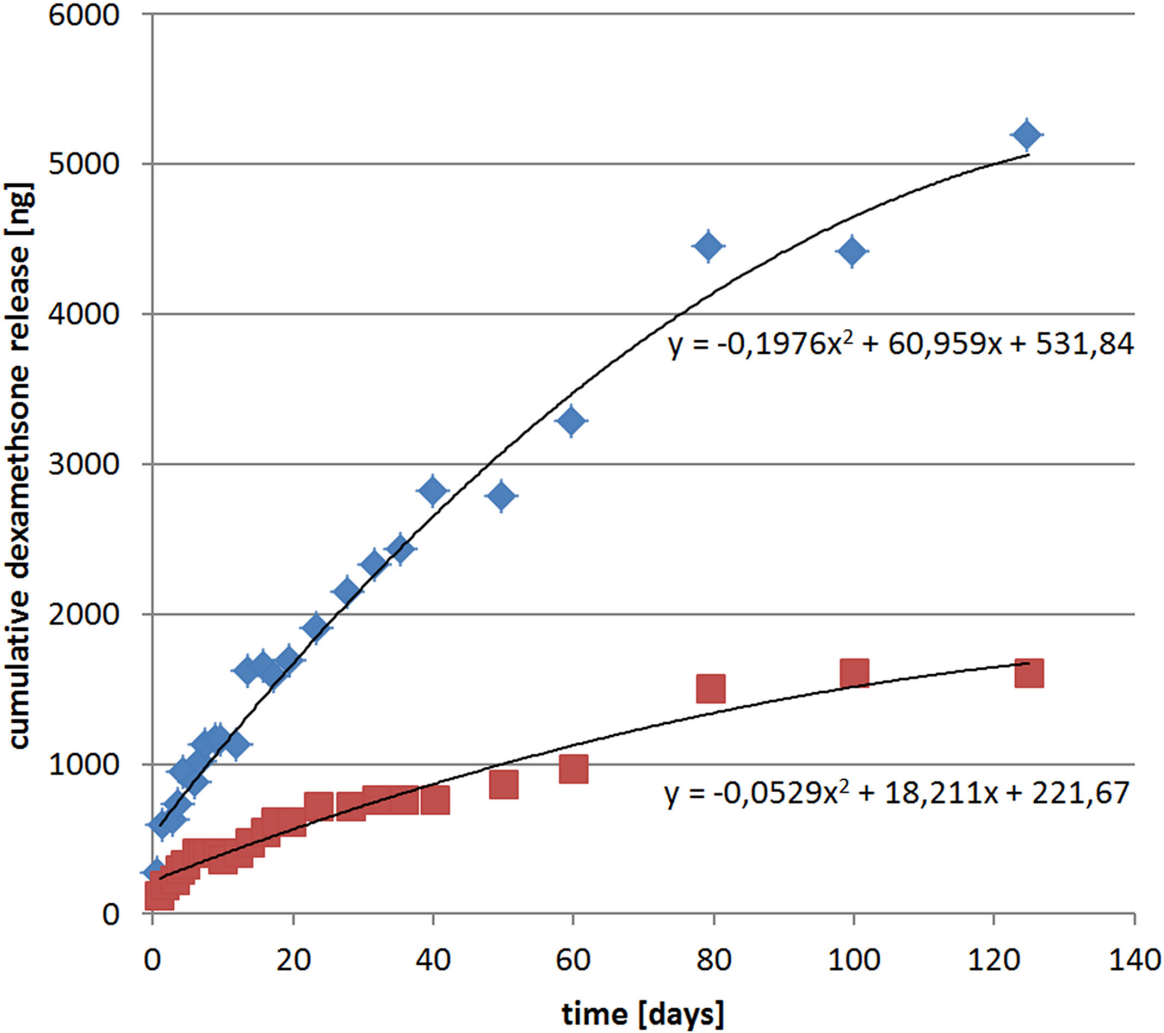
Dexamethasone release rates from guinea pig electrode measured in vitro. The first 4 mm of the electrode array were immersed into 1 ml of saline solution at 37°C. The saline solution was periodically sampled and DEX concentrations were measured by HPLC-MS (Agilent 1200 HPLC coupled with a Bruker MicrOTOF-Q II MS-detector). Representative release profile measurements for the 1% (red squares) and 10% (blue triangles) DEX loaded electrodes are shown. Over the experimental time period (91 days) the average release rate of DEX from the 1% and 10% loaded electrodes was estimated to be in the order of 16 ng/day and 49 ng/day respectively. DEX release rates were higher in the initial elution period (e.g. average over the first five days: 62 ng/day and 166 ng/day respectively for the 1% and 10% electrodes) with a slow decay over time.

The implant consisted of two flat active platinum contacts each welded to insulated platinum-iridium wires. The 1st contact was located more basal and the 2nd contact was located more apically on the tip of the electrode array with both wires being embedded in a medical grade silicone carrier. Control (0% DEX) and DEX (1% and 10%) electrodes are shown in Fig 1A–1C. Both active contacts as well as a reference ball electrode were linked to a percutaneous connector (Fig 2). DEX release rates from the silicone carrier were measured in vitro using HPLC-MS detection analysis (Fig 3). In view of the positioning of the electrode array in the cochlear (i.e. 3 mm entry via the RW with 1 mm adjacent) the first 4 mm of the electrode array were subject to release analysis (see Fig 3 for method details). Over the experimental time (91 days) DEX release rates in 1%- and 10%-loaded electrodes was estimated to average approximately 16 ng/day and 49 ng/day respectively. The release rates of DEX were higher in the early stages averaging 62 ng/day for the 1% electrode and 166 ng/day for the 10% electrode for the first 5 days with a slow decay over time (Fig 3).

### Acoustically Evoked Auditory Brainstem Response (AABR)-measurement

AABRs were recorded prior to surgery (day 0) to examine the baseline hearing threshold and again after surgery on days 0, 7, 28, 56, and 91 just before euthanasia. Measurements were performed using the system set-up of Tucker Davis Technologies (TDT, Alachua, Florida, USA). Recordings were performed using four subcutaneous needle electrodes; sinusoidal signals being generated with 10 ms duration. Acoustic tone stimuli were determined in 10 dB steps from 0–70 dB SPL with frequencies of 1 kHz, 4 kHz, 8 kHz, 16 kHz, 32 kHz and 40 kHz or, in animals hard of hearing, from 30–90 dB SPL. Further detailed information of this methodology can be found in [18].

### Surgery

In all animals the left ear was implanted whereas the right cochleae were implanted in 6 animals only of the 0% DEX group; (i.e. control electrodes were bilaterally implanted in these animals). Animals were shaved behind the outer ear and on the vertex, the exposed skin area was disinfected and local anaesthesia was applied. To fix the implant connector on the skull, the bone was roughened using a diamond drill and four screws were anchored in the bone using (poly)methyl-methacrylate. Subsequently the bulla was opened to expose the cochlea, the round window membrane was then incised and the implant inserted into the basal turn of the scala tympani, up to and including the spotted 3 mm mark.

To intensify the implantation trauma the implant was inserted three times and removed twice. Additionally, a muscle graft was used to close the round window and to increase tissue reaction [22].

The electrode lead wire was fixed at the bulla using (poly)methyl-methacrylate, the muscles were adapted and the skin was sutured close. The weight, signs of potential pain and surgical wounds of the animals were clinically monitored for five days and subsequently weekly.

### Impedance Measurements and Electrical Stimulation

Animals were stimulated using a standard MED-EL PULSARci100 stimulator with a HD-CIS 750 pps coding strategy to generate biphasic monopolar pulse trains with a charge of 16 nC. The default stimulation amplitude was 453 μA and the default phase duration was 33.75 μs. If for high impedances (Z) the compliance limit of the stimulator was reached, the phase duration was increased and the stimulation amplitude decreased accordingly to realise the desired stimulation charge of 16 nC.

Impedance was measured following surgery and was carried out weekly until day 91, prior to animal sacrifice. Z-measurements were taken before electrical stimulation (ES) starts, after 10 minutes, 30 minutes and 60 minutes of ES. Starting with the first (basal) contact for each time point, impedance was measured three times, followed by three subsequent impedance measurements at the second contact (apical). For data analysis the mean of the three measurements were taken for each contact. Impedance measurements were performed at 16.67 kHz using a MED-EL Cochlear-Implant-System and MAESTRO Software 4.0. The measurement frequency of 16.67 kHz is based on a typical stimulation burst with a pulse phase duration of 30 μs. The MED-EL Cochlea-Implant-System is composed of a PC, the Diagnostic Interface Box II (DIB II) and the speech processor OPUS.

### Histology

Following the last impedance measurement on experimental day 91, all animals were euthanized by transcardial perfusion with phosphate-buffered saline and paraformaldehyde. Temporal bones were removed and prepared for epoxy-embedding and grinding as described previously [18]. Briefly, the oval window and the apex were opened and the cochlea was immersed in Wittmark fixative solution for 24 hours. Afterwards the specimens were rinsed in lithium sulphate for 10 hours, dehydrated in alcohol and dried at room temperature. Specimens were embedded in epoxy resin and mixed with titanium oxide under vacuum. Grinding level of epoxy resin specimens was documented every 20 μm, each level then being stained with toluidin-blue and eosin-orange. Images were taken at 30-, 150-, 200-, and 400-fold magnifications with a digital microscope equipped with image-analysis software (VHX-600, Keyence).

Since connective tissue was observed only in the first turn of the cochleae, the tissue span was evaluated for three zones of the basal scala tympani, these being the areas of the round window niche (RWN), the basal region behind the niche and the first turn behind the modiolus. To calculate the percentage of fibrous tissue growth, the area of the scala tympani was accessed by tracking the inner outline of the scala tympani using Keyence software. From this outlined section, the part occupied by the electrode was subtracted to obtain the free area of the scala tympani which could potentially be filled with connective tissue. This area was normalized as 100%. The area filled with new tissue was identified using the same Keyence system and software as mentioned above. The percentage of scala tympani filled with connective tissue was calculated in relation to the selected normalized area (100%).

### Statistical evaluation

Impedance and AABR analysis as well as tissue growth were analyzed for significance using the Shapiro-Wilk normality test and subsequently, due to an absent Gaussian distribution, the Bonferroni multiple comparison test (non-parametric multiple comparison with α-correction). Potential correlations between tissue growth and hearing loss as well as that between impedance and tissue growth were analysed using the Spearman-Rho non-parametric correlation test. In all tests differences with α < 0.05 were considered as significant.

## Results

### Impedance

All electrodes, tested in saline solution pre-operatively, were found to have impedances below 4 kΩ. In animal groups, impedances on both basal and apical contacts were measured before and after 60 minutes of electrical stimulation on days 0, 7, 28, 56 and 91. On day 0, impedances were between 5 to 6 kΩ post-operatively in all groups at each electrode.

**Fig 4:**
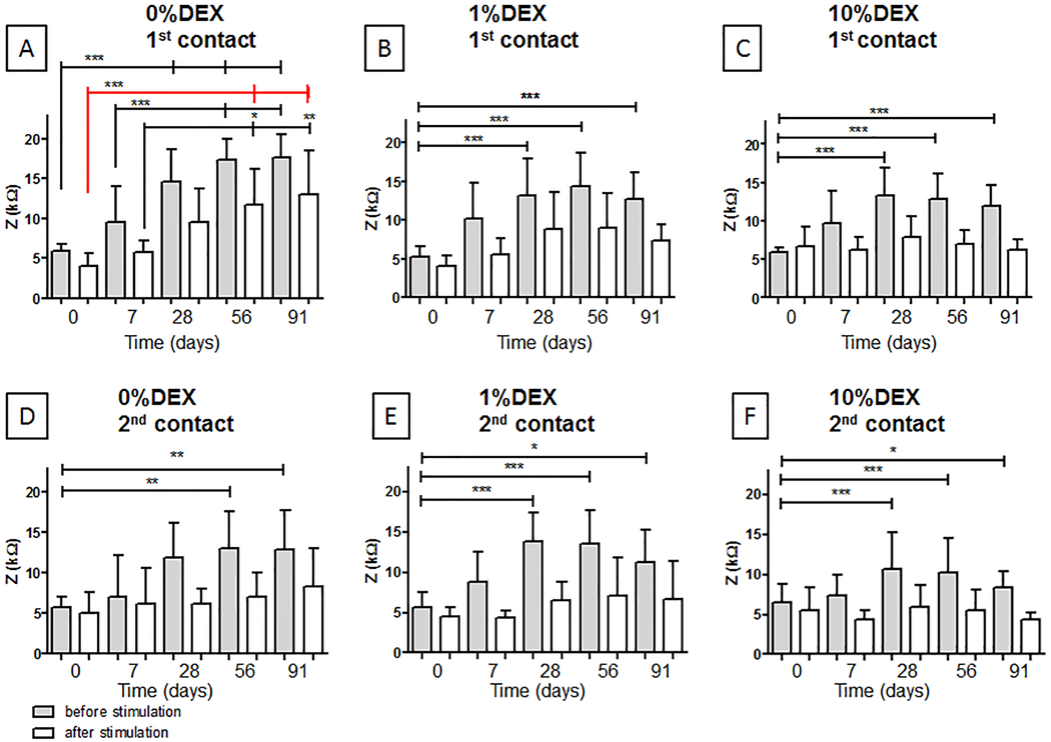
Mean impedance (y-axis) before and after 60 minutes of ES are plotted for all measurement days (x-axis). Sections A-C show the impedances at the first contact (basal, near to the RWN) for each experimental group whereas sections D-F visualize the impedances at the second, more apical, contact. Statistical differences in impedances between the experimental days were denoted: * = p<0.05; ** = p<0.01; *** = p<0.001; error bars = SD.

On the unstimulated basal contact, impedances increased significantly (p<0.001) over time from the day of implantation until day 91 in all experimental groups (Fig 4A–4C, grey bars). One week after implantation impedances on the unstimulated basal contact were slightly increased although this effect was not statistically significant. In contrast, on day 28 impedances of all experimental groups were significantly higher (p<0.001) compared to those measured on day 0 (0% DEX: mean of 14,57 kΩ; 1% DEX: mean of 13,15 kΩ; 10% DEX: mean of 13,27 kΩ). From day 28, impedance measurements of the unstimulated basal contact from all three groups did not change significantly.

**Fig 5:**
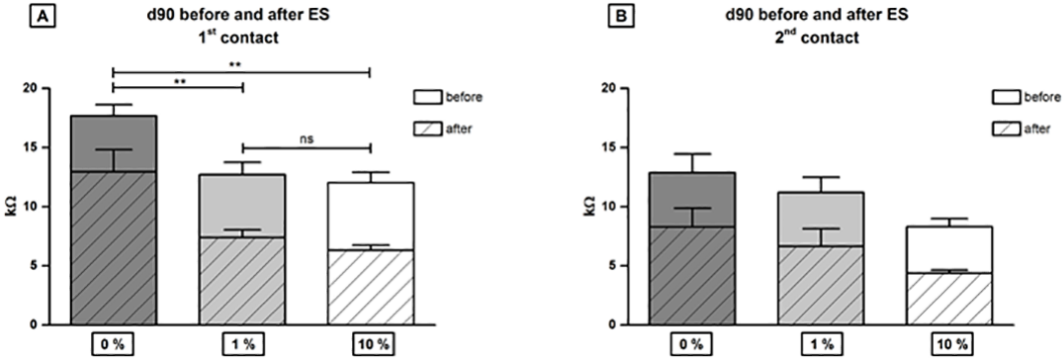
Impedances of contact one (A) and contact two (B) in all three treatment groups before (clear) and after (dashed) electrical stimulation on experimental day 91. At contact one (A) in both DEX treated groups impedances were significantly reduced after 3 month observation time compared to the group receiving 0% DEX (** = p<0.01) and there was no difference between impedance levels in 1% DEX or 10% DEX treated individuals detected at contact one (ns = not significant). At the second contact, which was located more apical in the cochlea, no significant differences in impedance values between treatment groups were detected (B). Error bars = SEM.

In both DEX treated groups impedances were significantly reduced after the 3 month observation time compared to the 0% DEX group. However, there were no statistical differences between the impedance levels measured in the 1% DEX or 10% DEX treated animals (Fig 5A).

With regard to the apical contact (contact two), increased impedances were detected in both unstimulated 0% DEX and 1% DEX animals (Fig 4D and 4E). In contrast, increased impedance was not observed in unstimulated contacts in the 10% DEX group (Fig 4F). Impedances at both the unstimulated apical 0% DEX and 1% DEX contacts increased until day 28. Significant increases in impedance were observed for the 1% DEX group on days 28, 56 and 91 compared to day 0 (Fig 4D and 4E). No differences in impedance levels on the apical contact were detected after 3 month implantation before or after electrical stimulation (Fig 5B).

No impedance increase over time was detected after stimulation (Fig 4, white bars) at each electrode contact in each group but the basal contact of the control group receiving 0% DEX (Fig 4A). In this group impedances on experimental days 56 and 91 were significantly increased after electrical stimulation compared to day 0 impedance (Fig 4A, red line; p<0.001).

In general, impedances were higher on the 1^st^ contact, in the region of the round window niche, and lower at the 2^nd^ contact, which was located more apical in the implanted cochlea. After the start of ES, a decrease in impedance was observed in all groups compared to the prior unstimulated levels. The highest impedances were measured on basal contacts in the 0% DEX group before and after electrical stimulation.

### Residual hearing preservation

**Fig 6:**
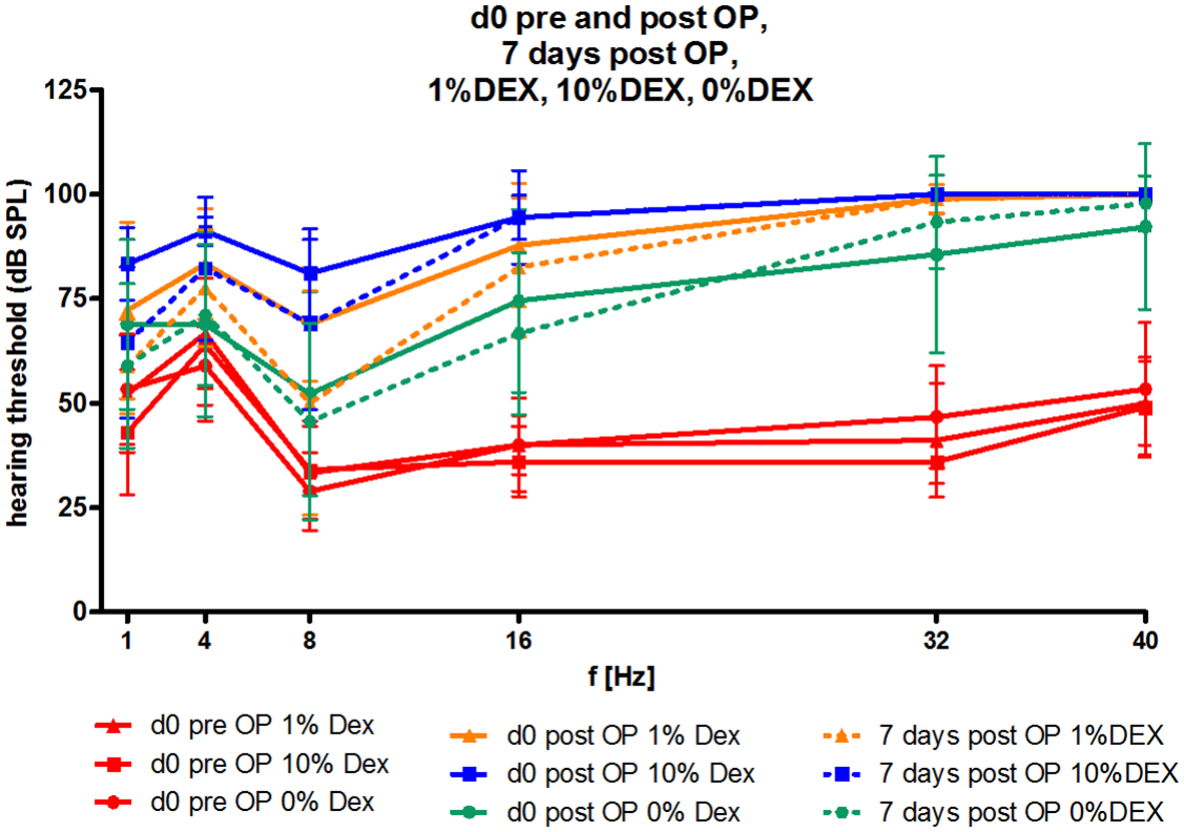
Hearing thresholds on experimental day 0 before and after implantation and on day 7. The mean and SD hearing threshold (dB SPL) in the frequencies tested (1–40 kHz) did not differ between groups on experimental day 0 before or after implantation, or on day 7. The hearing threshold in all experimental animals before insertion of the electrode array was equal (red lines). Directly after implantation (straight lines) the hearing threshold increased in all groups and did not change until experimental day 7 (dotted lines).

**Fig 7:**
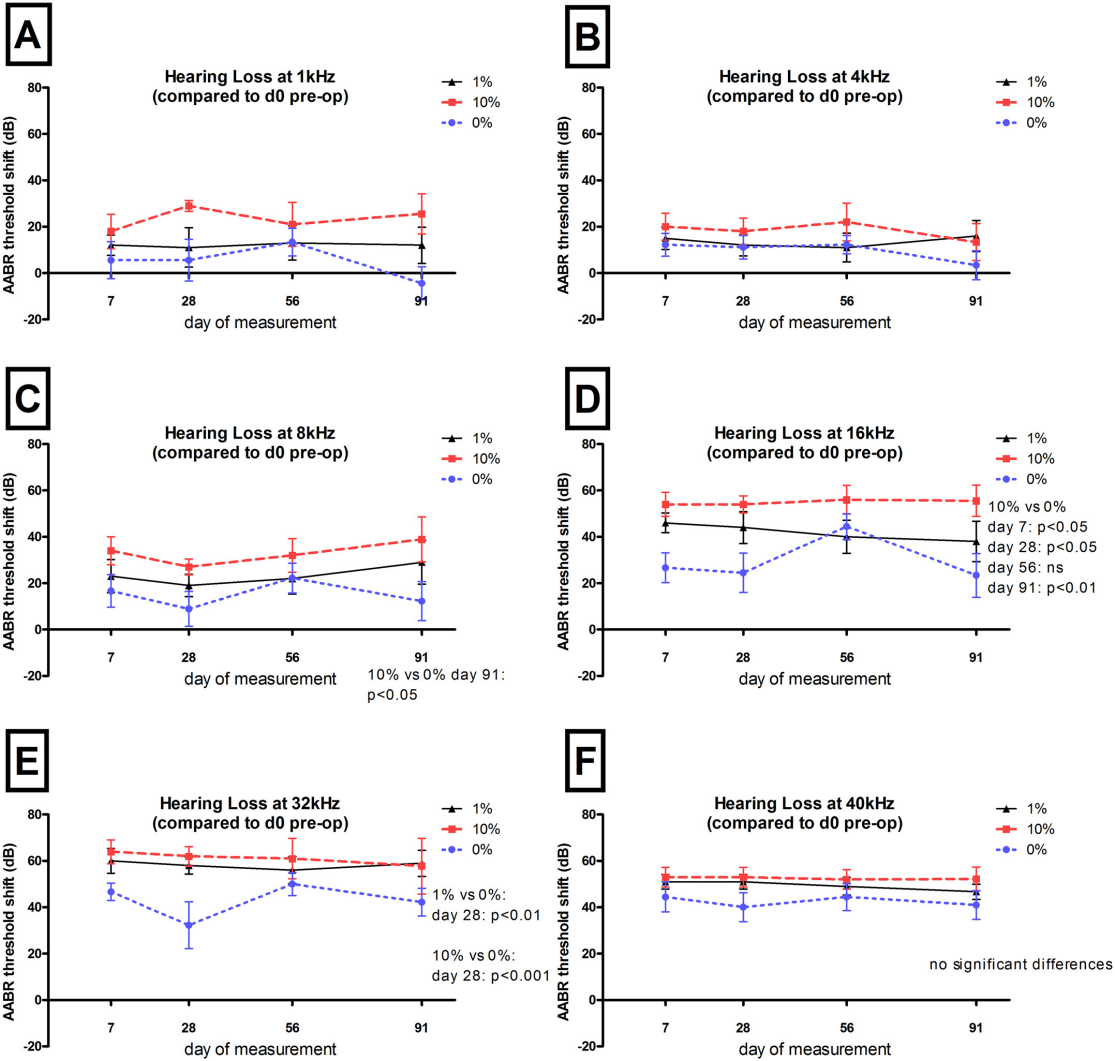
AABR threshold shifts related to day 0. The mean and SEM AABR threshold before electrode insertion is plotted as a function of time. Significant differences in hearing thresholds are listed in the respective graphs. DEX eluting electrodes did not protect residual hearing at any frequency. At the lowest frequencies (1 and 4 kHz) and at the highest frequency tested (40 kHz), no significant differences were detected between the groups. At 8, 16, and 32 kHz DEX treated animals had an increased hearing loss compared to control (0% DEX).

AABR measurement were analysed on days 0 (pre- and post-operative), 7, 28, 56, and 91. All animals included in this study were with normal hearing (Fig 6). The electrode insertion trauma (EIT) induced by the three-time insertion and twice removal of the electrode resulted in hearing loss in all animals of each experimental groups. No significant differences between groups directly after surgery or 7 days after surgery were observed (Fig 6). Electrode insertion produced hearing loss (threshold shift) between 5 to 30 dB in the lower ((1–8 kHz) frequencies (Fig 7A–7C) and between 20 to 60 dB in the higher (16–40 kHz) frequencies (Fig 7D–7F) as measured on day 7.

No significant differences in hearing thresholds are detected in the very low frequencies (1 and 4 kHz) or at the highest frequency tested (40 kHz) between all groups (Fig 7A, 7B and 7F). At 8 kHz and 16 kHz, animals treated with 10% DEX had significantly reduced hearing on day 91 compared to control (0% DEX) animals. In the 16 kHz region this difference was already visible at day 7 post-surgery. At 32 kHz no differences were observed in all groups 91 days after surgery although at this same frequency significant increases in hearing loss on day 28 for both the 1% DEX (p<0,01) and 10% DEX groups (p<0.001) were observed (Fig 7E).

**Fig 8:**
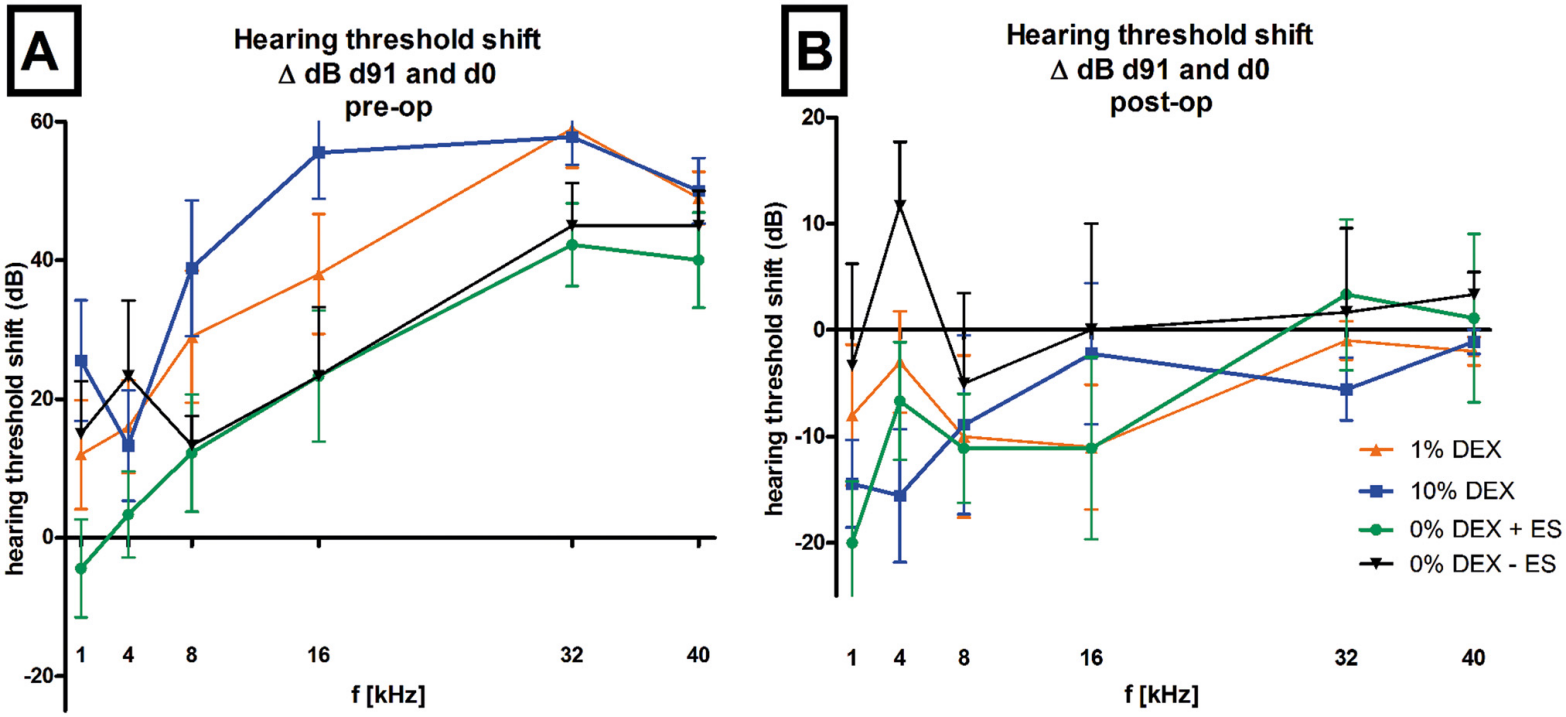
Shifts in hearing thresholds obtained on day 0 before (A) and after surgery (B) until day 91 are plotted (ΔdB) for all tested frequencies. At 4, 8, 32 and 40 kHz no differences in ΔdB between treatment groups were observed for day 0 (pre-operative) and day 91. 10% DEX treatment resulted in significantly greater threshold shifts at 1 and 16 kHz than 0% DEX + ES (p<0.05) or 0% DEX without-ES at 16 kHz (p<0.05) (A). No differences between groups where observed when relating the final threshold (day 91) to the day 0 threshold measured after surgery (B). Error bars: SEM.

Comparing the hearing threshold shifts of the three experimental groups on day 91, no protective effect on EIT was detected for both DEX treatments, neither when taking the pre-operative thresholds (Fig 8A) nor when comparing the post-operative thresholds on day 0 (Fig 8B). No differences were observed when relating AABR data obtained on day 91 to that directly measured after surgery. However, relating AABR data obtained before surgery to that measured on day 91, significant differences can be detected between the hearing thresholds of 10% DEX treated animals and 0% DEX animals (+ES) obtained at 1 kHz (p<0.05) and 16 kHz (p<0.05) and at 16 kHz for 10% DEX versus 0% DEX (without ES), DEX treated animals would appear to be suffering from a higher hearing loss than controls (0% DEX).

### Fibrous tissue growth

**Fig 9:**
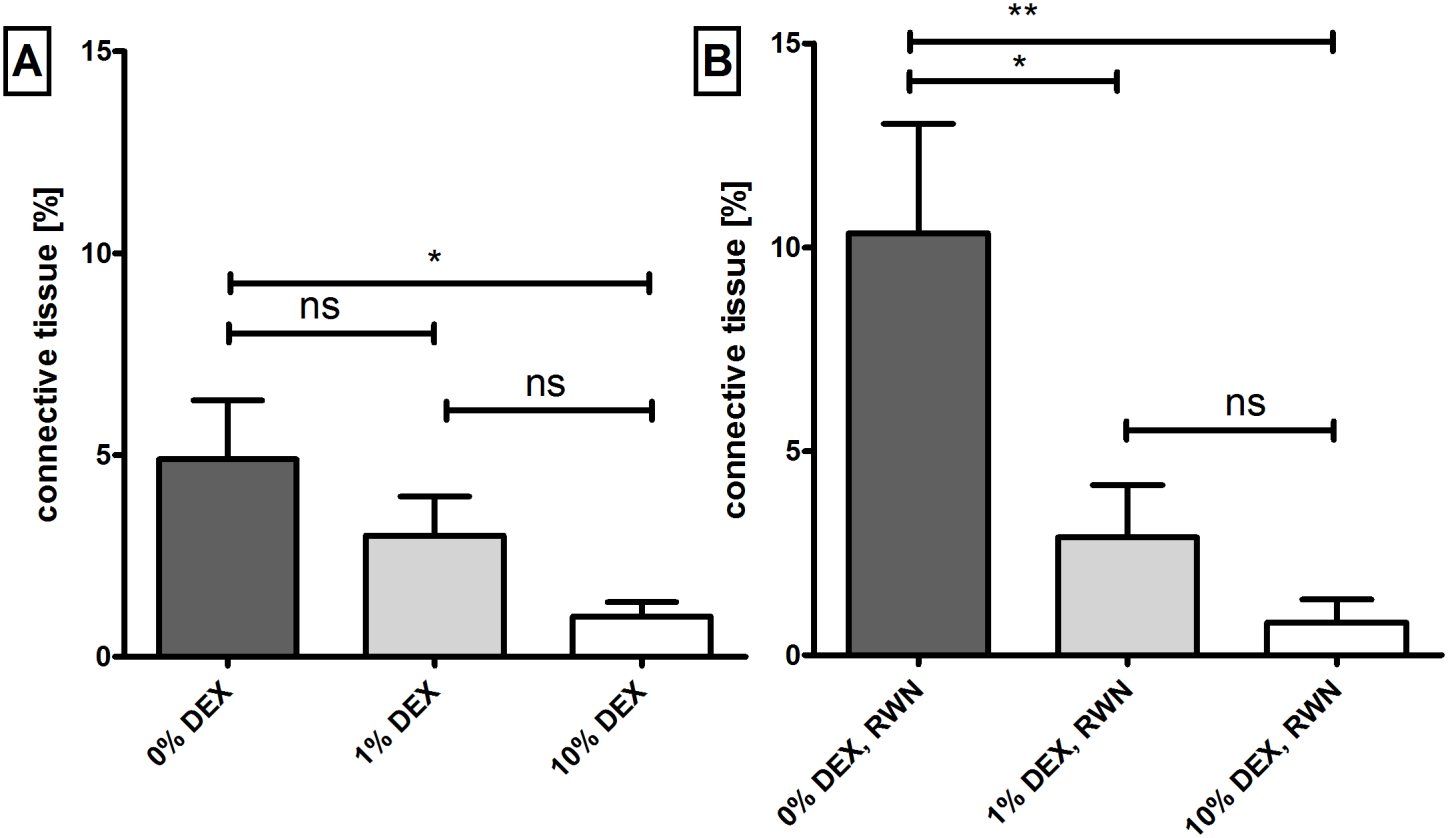
Connective tissue formation in the scala tympani of implanted animals treated with 1%, 10% or 0% DEX (control). Tissue growth is plotted as the percentage area filled in the scala tympani. Sector A illustrates the mean tissue growth for the whole cochlear length analyzed (basal turn) showing a significant difference between the 10% DEX treated cochleae and 0% DEX (* = p<0.05). Comparing the tissue reaction at the RWN (B), significant differences are observed for both DEX groups compared to 0% DEX. Statistical differences were denoted; ** = p<0.01; ns = not significant. Horizontal bars represent the SEM.

**Fig 10:**
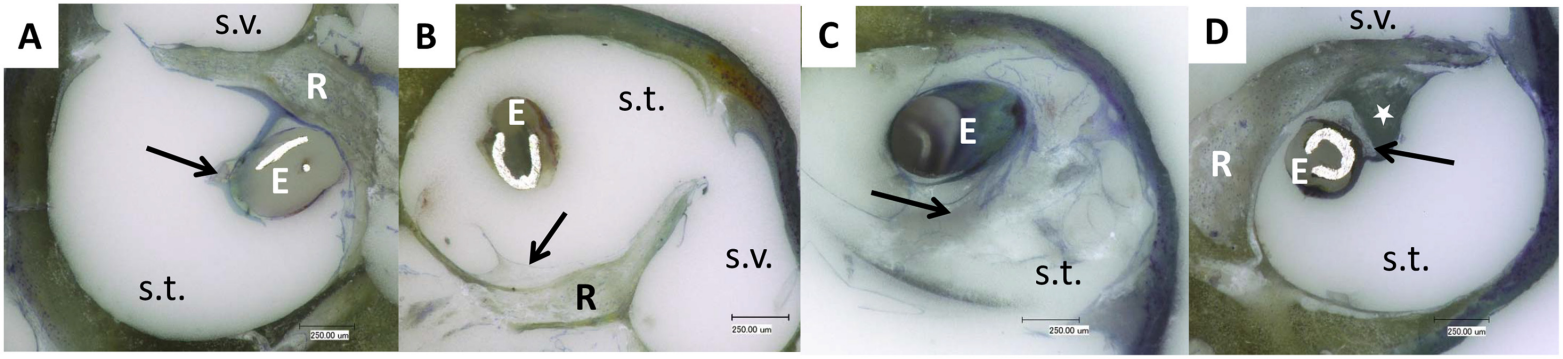
Representative images of grinded cochleae. The tissue reaction in 10% DEX (A), 1% DEX (B), 0% DEX + ES (C) and 0% DEX without ES (D) treated guinea pig cochleae is representatively shown. E: electrode; s.t.: scala tympani; s.v.: scala vestibuli; arrow: fibrosis; star: ossification; R: Rosenthal‘s canal; Magnification: 150fold.

Cochleae treated with 10% DEX had significantly (p<0.05) reduced areas of connective tissue compared to 0% DEX cochleae (Fig 9A). A reduction approaching 80% was observed (0.99 ± 0.36% versus 4.89 ± 1.45%; mean ± SEM). In contrast, tissue growth was reduced by approximately 40% (3.00 ± 0.96%; mean ± SEM) in 1% DEX treated cochleae although this decrease was not statistically significant (Fig 9A). However, as shown in Fig 9B, by focusing on the region of the RWN it was observed that fibrous tissue formation was reduced significantly by more than 70% and 90% respectively with the 1% DEX (2.88 ± 1.28%; mean ± SEM; p<0.05) and 10% DEX (0.80 ± 0.56; mean ± SEM; p<0.01) treatments compared to non-DEX treated cochleae (10.35 ± 2.68%; mean ± SEM). No statistical differences were seen between the 1% and 10% DEX treated groups. No effect of administering 60 minutes short-term electrical stimulation per week was detected on connective tissue growth along the whole area analyzed (S1 Fig). Representative images of fibrosis detected in the experimental groups are shown in Fig 10).

### Correlation between fibrous tissue growth and hearing loss

**Fig 11:**
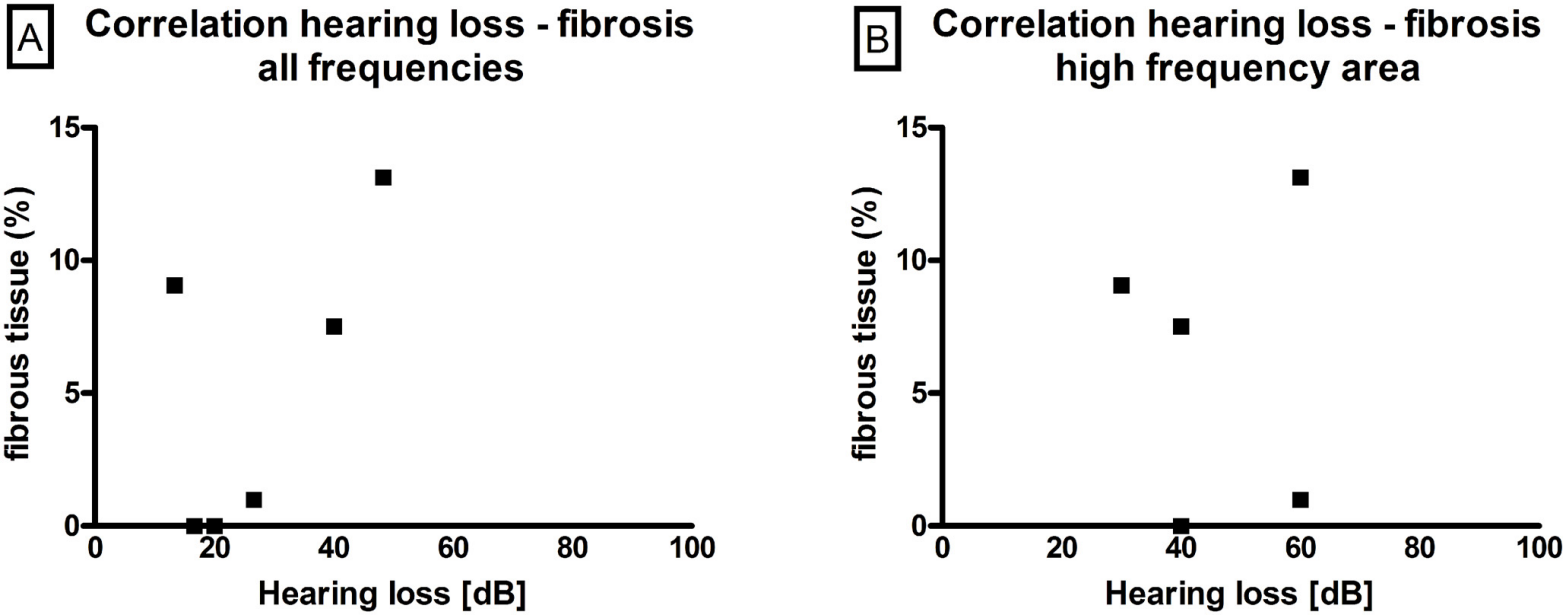
Correlation between fibrous tissue formation and hearing loss. No correlation between fibrous tissue growth and hearing loss, neither for all frequencies tested (1, 4, 8, 16, 32 and 40 kHz; A)) nor for the high frequencies (32 and 40 kHz; B) were observed in ears implanted with a 0% DEX-electrode array (no-electrical stimulation).

To evaluate the potential correlation between hearing loss and fibrous tissue growth, hearing data and histology of each individual animal implanted with a 0% DEX electrode (no-electrical stimulation) was analysed. Firstly, the mean hearing loss of each animal (i.e. the difference between hearing thresholds on day 91 and day 0 before surgery) of all frequencies (Fig 11A) was compared with the relevant fibrous tissue growth detected in the three zones analysed (i.e. the areas of the round window niche (RWN), the basal region behind the niche and the first turn behind the modiolus). Using the Spearman-Rho non-parametric correlation test, no correlation was observed. When comparing the hearing loss of the high frequencies (32 and 40 kHz), which correspond anatomically to the area being analysed histologically, no relevant dependency between hearing loss and fibrous tissue growth was again observed (Fig 11B).

### Correlation between fibrous tissue growth and impedance

**Fig 12:**
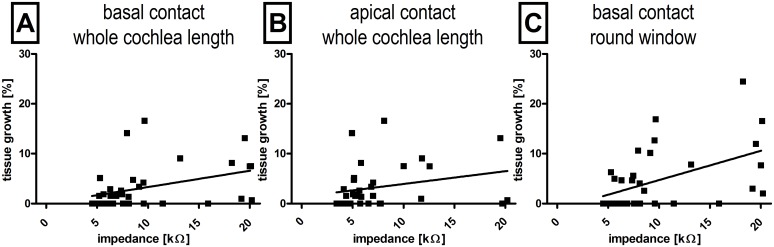
Correlation between tissue growth and impedance. Tissue growth around the electrode array significantly correlated with the measured impedance on the basal electrode (A) as well as on the more apical electrode (B). The highest correlation was found when the tissue growth at the round window was compared to the impedance measured on the basal contact (C).

The potential correlation between impedance and fibrous tissue growth at the 3 months post-surgical time-point was investigated using the Spearman-rho statistical test. Positive correlations between impedance increases over time and fibrous tissue growth around the implant at electrode were observed for the more basal located contact one (r = 0.37; p = 0.031) as well as the more apical contact two (r = 0.38 with p = 0.024). Additionally, tissue growth in the region of the RWN correlated with the measured impedances (r = 0.48 p = 0.0036). Impedances were measured after the last set of electrical stimulation directly before animal sacrifice. The mean data of each animal in each experimental group is shown in Fig 12.

## Discussion

Fibrous tissue growth after cochlear implantation is one factor that may diminish the performance of the device in patients. In this study we were able to show that A) there is a positive correlation between fibrous tissue growth and impedance increase; B) localized DEX treatment reduces electrode impedance and fibrosis and C) the said drug delivery system allows local administration of a drug entity such as DEX at therapeutically relevant concentrations for sufficient time duration.

### Fibrous tissue growth and impedance increase

The electrode trauma produced from the multiple insertions and withdrawal procedure of the array into the cochlea generated an appropriate tissue reaction seen in all experimental groups allowing the potential correlation of fibrous tissue growth and impedance changes to be investigated. In this model fibrous tissue growth in the 0% DEX group is limited to the basal turn of the cochlea with 4.89% of the free scala tympani area (i.e. scala tympani area less the area of the electrode array itself) occupied with new tissue development. The amount of connective tissue deposition is more extensive at the RWN with 10.35% of the area covered. Since it is well considered that impedance provides information on the status of the electrode–tissue interface, the aim of this study was to evaluate whether a correlation exists between the level of fibrotic material and impedance. Feasibly, such correlation may help to provide predictive information concerning the levels of fibrosis in the scala tympani of cochlear implant patients.

A limited number of studies in vitro focused on cochlear implantation have investigated the inflammatory reaction and subsequent development of fibrotic plaques [23–27]. At the same time, a conspicuous absence of relevant models in vivo investigating these phenomenon and the underlying mechanisms has been notable. To date, the signaling pathways responsible have been partly elucidated using treatment strategies against general inflammatory reactions such as glucocorticoids or JNK inhibitors [28] as well as from descriptions of the general tissue reactions following implantation [29]. Clinically, local delivery of corticosteroids during surgery has been associated with reduced electrode impedance suggesting that this drug class may influence the tissue response to the foreign body response and indirectly suggest a correlation between fibrosis and electrode impedance [9, 17, 30]. While Xu et al (1997) demonstrated that the degree of spiral ganglion cell loss is correlated to both the degree of inflammation and electrode insertion trauma [5], no study to date has proven a direct correlation between impedance and the level of fibrosis.

By relating histological and electrophysiological data we have provided evidence that measurement of impedance can render direct information on the status of tissue formation in the scala tympani. As a consequence, we suggest that electrode impedance may be a useful diagnostic tool to estimate the extent of fibrous tissue formation in patients who have received cochlear implants especially since no direct assessment of fibrosis is currently possible. As well as being non-invasive, impedance measurements may be useful in studies evaluating drug action and duration since they are very rapidly accrued (seconds to minutes) depending on the number of contacts and repetition rate and allow similar sequels over time.

As observed previously by Charlet de Sauvage et al. [31], the present study saw that electrode impedance decreased after electrical stimulation compared with pre-stimulus levels. However, overall impedance at the more basal contact increased over time in the 0% DEX group (contact one, Fig 1A) in the presence and absence of electrical stimulation although further impedance elevations was not observed after 4 weeks, a similar finding to that of Paasche et al. [9]. Recently, Newbold and colleagues detailed the nature of the events responsible for impedance decrease following electrical stimulation [8]. The total electrode impedance (Z_t_) can be broken down into access resistance (R_a_) and polarization impedance (Z_p_), where R_a_ represents the resistance of the electrolyte and its contents around the electrode and Z_p_ includes the resistance and capacitance of the double layer at the electrode surface and the corresponding Faradaic impedance across this layer [32]. While a fibrous tissue matrix covering may lend towards fostering higher R_a_ and Z_p_ values, stimulation-induced changes are likely to be confined to the electrode interface. Newbold et al. (2014) demonstrated that for chronically implanted and stimulated cochlear implant electrodes electrical stimulation caused a transitory reduction in impedance values, an effect that can be observed several years after implantation [8]. The majority of impedance reduction occurred via a reduction in polarization impedance, Zp. These results suggest that stimulation affected the electrode surface rather than creating change in the fluid and tissue of the scala tympani. Possible changes that could be occurring are protein adsorption and desorption and/or a reduction in adhesion of the fibrous tissue matrix to the surface of the electrode [8].

### Dexamethasone effect on tissue reaction, hearing and impedance

Animal studies have shown that a single application of DEX at the time of implantation reduces fibrous tissue growth [21, 33, 34]. Other studies using DEX-eluting arrays have shown a significant reduction in inflammatory cells after 13 days [35], and reduced fibrous tissue levels measured 28 days after implantation [16, 18]. However, longer term studies over 3 months have not seen any significant effects on fibrous tissue growth [20, 36]. In the present study the fibrous tissue area measured 3 months post-surgery was reduced in animals that received DEX-eluting cochlear implants (1% and 10% DEX).

The effect of corticosteroids, in particular DEX, on cochlear implantation related loss of residual hearing has been studied extensively. A number of studies have observed hearing protection where DEX was given prior to or after surgical implantation either by systemic administration [21, 33] or locally on the round window or into the scala tympani [18, 21, 28, 34, 36–39]. Stathopoulos et al. [36] proposed that the elution rate of the steroid from the implant as well as the degree of trauma inflicted during the surgery were possible factors that could influence efficacy. Importantly, the same study saw that DEX dosages used in the study were safe in the guinea pig cochlea and did not retard the development of the cochleostomy seal [36]. The electrode trauma model used in this study saw an initial threshold shift in all experimental groups for every tested frequency with moderate hearing loss in the lower frequencies (1, 4, 8 kHz) and being more extensive in the higher frequencies (16, 32, 40 kHz; Fig 6). Measured the day directly after surgery (day 0 post OP), increased hearing thresholds of the lower frequencies (1–16 kHz) decreased in all experimental groups within the first week of implantation. The hearing threshold increase after surgery is, amongst other reasons, based on structural damage caused by the multiple insertion and withdrawal of the electrode in the model utilised. Depending on the extent of damage, a sufficient level of fibrosis can be promoted as well as hearing loss. The effect of residual hearing loss due to CI implantation is well known and multifactorial [40] and a decrease of hearing threshold directly after surgery until day 7 after implantation may be due to resorption of air and a rearrangement of cochlear homoeostasis. Interestingly, the 10% DEX group showed the highest hearing threshold in the lower frequencies when compared to the other experimental groups. This discrepancy started directly after surgery and did not change over time. Three months after implantation hearing thresholds in the 10% DEX treated animals were higher than 0% DEX animals with electrical stimulation at 1 and 16 kHz or than 0% DEX animals without electrical stimulation at 16 kHz (Fig 8). Since directly after surgery the hearing threshold of the 10% DEX group was more extensively increased than those of the other groups, it was speculated that the actual physical loading of the DEX on the electrode may have been the reason behind this elevated hearing loss. Certainly from the manufacturing process the 10% DEX electrodes are not stiffer in constitution than those loaded with 1%, thereby negating this parameter as a causative factor for the higher trauma levels found in these animals. Feasibly the concentrations of DEX released by the 10% loaded electrode may have produced local cellular damage in the cochlea. A recent study by Liu et al (2015) has shown that in guinea pig, burst-release concentrations of 10% DEX electrodes can attain micromolar levels (6.2 μM at 30 minutes) although this is transient falling to sub-micromolar concentrations within 24 hours (0.93 μM) [41]. Whether or not these burst-release concentrations of DEX can promote cellular damage to the cochlea is speculative. However, studies by Piu et al (2011) [42] using OTO-104, a poloxamer-based hydrogel containing micronized dexamethasone for intratympanic (IT) delivery in guinea-pig, observed burst-release levels of DEX in the perilymph to be up to 40-fold higher than those obtained by Liu and colleagues (2015) using the 10% DEX loaded electrodes and without provoking any major effects on hair cell pathology or inducing hair cell loss.

Fibrosis has been proposed to be a factor reducing residual hearing [43]. We observed that fibrosis was reduced with both DEX treatments in the area of the round window niche but hearing in high frequencies was not preserved, indicating, at least in this case, a lack of correlation between new tissue growths and hearing thresholds. The possibility that surgical traumas were higher in one group compared to another were analyzed by calculating ΔdB hearing thresholds at day 91 and those from post-surgery thresholds on day 0 for each individual animal. No differences between the treatment groups were observed. Fibrous tissue growth after cochlear implantation manifests itself due to trauma either from electrode insertion or acoustic and vibratory trauma from drilling, foreign body reaction or even from tissue displacement of bone dust or blood into the perilymphatic space. This new tissue formation interferes with the flow of electrical charge from the electrodes to the spiral ganglion neurons, thereby increasing the power requirements of the cochlear implant [8]. Feasibly, the change of electrode impedance over time can be used as a non-invasive indirect estimation for intracochlear fibrous tissue deposition in human patients. Pharmacological modification and prevention of fibrous tissue growth after cochlear implantation is likely to be dependent on the dosing regimen (e.g. single administration, prolonged release from drug delivery system), timing of the administration (e.g. prior to drilling of the cochleostomy/ round window membrane opening), the choice of drug and its physical/biological characteristics as well as the fibrosis model itself. Together, these variables potentially influence the outcome of studies investigating therapeutic treatments for attenuating fibrosis. To date, no model has been exemplified or proposed as the standard to be used by investigators in the various laboratories and possibly as a consequence of this fact, reporting from the studies has been understandably diverse in nature.

## Conclusion

Using the above described conditions DEX significantly reduced impedances and fibrous tissue growth after 3 month observation. The fibrous tissue growth detected after 91 days of implantation did not correlate to the detected hearing loss but did significantly correlate with the impedance increase over time. Therefore impedance data may give a reliable idea of fibrous tissue growth around the electrode array *in vivo*. The incorporation of DEX into the electrode arrays silicone is a sufficient drug delivery method to apply a biologically active amount of DEX into the scala tympani of the cochlea. This DEX eluting CI is a promising device for impedance and fibrosis reduction in cochlear implant patients.

## Supporting Information

S1 Fig Effect of short term electrical stimulation on fibrosis. No effect of 60 minutes electrical stimulation per week was detected on connective tissue growth along the whole area analysed (left graph). In the region of the round window niche (RWN, right graph) electrical stimulation seems to have increased the tissue response (bar 0%DEX), leading to more connective tissue growth around the implant in that area. * = p<0.05; ** = p<0.01; ns = not significant; error bars = SEM.
(PDF)
