# Supplementary material for: Impedance Changes and Fibrous Tissue Growth after Cochlear Implantation Are Correlated and Can Be Reduced Using a Dexamethasone Eluting Electrode
Source: PLoS One. 2016 Feb 3;11(2):e0147552. doi: 10.1371/journal.pone.0147552 (PMC4739581; doi:10.1371/journal.pone.0147552)

SUPPORTING INFORMATION

S1 Fig. Effect of short term electrical stimulation on fibrosis. No effect of 60 minutes electrical stimulation per week was detected on connective tissue growth along the whole area analysed (left graph). In the region of the round window niche (RWN, right graph) electrical stimulation seems to have increased the tissue response (bar 0%DEX), leading to more connective tissue growth around the implant in that area. *=p<0.05; **=p<0.01; ns=not significant; error bars = SEM.

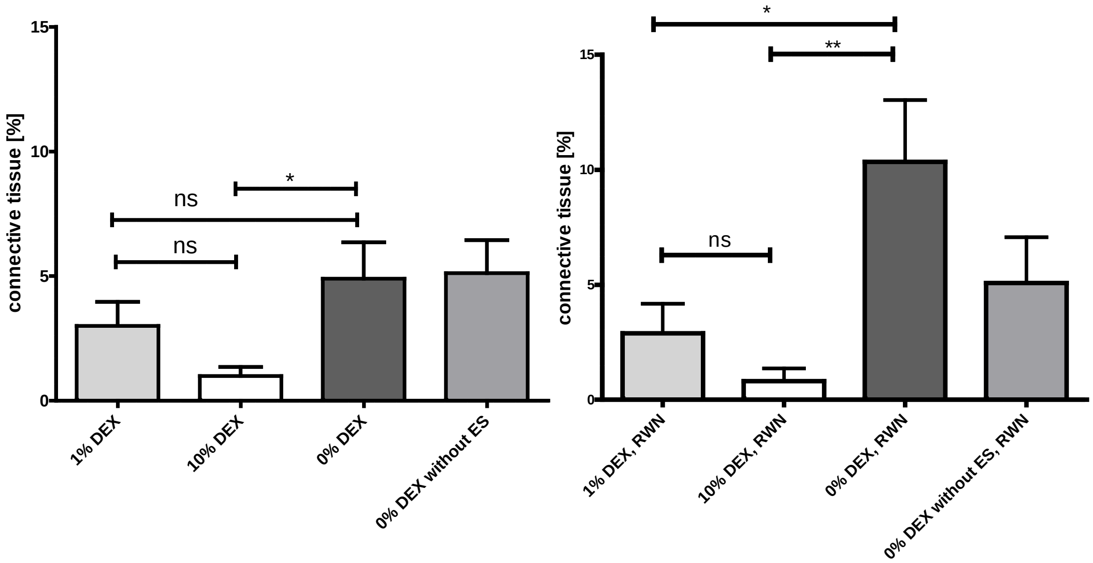

Supplement: S1 Fig — No effect of 60 minutes electrical stimulation per week was detected on connective tissue growth along the whole area analysed (left graph). In the region of the round window niche (RWN, right graph) electrical stimulation seems to have increased the tissue response (bar 0%DEX), leading to more connective tissue growth around the implant in that area. * = p<0.05; ** = p<0.01; ns = not significant; error bars = SEM. (PDF) [file pone.0147552.s001.pdf]
